# Sensor Fusion of Gaussian Mixtures for Ballistic Target Tracking in the Re-Entry Phase

**DOI:** 10.3390/s16081289

**Published:** 2016-08-15

**Authors:** Kelin Lu, Rui Zhou

**Affiliations:** School of Automation Science and Electrical Engineering, Beihang University, Beijing 100191, China; zhr@buaa.edu.cn

**Keywords:** sensor fusion, Gaussian mixtures, target tracking

## Abstract

A sensor fusion methodology for the Gaussian mixtures model is proposed for ballistic target tracking with unknown ballistic coefficients. To improve the estimation accuracy, a track-to-track fusion architecture is proposed to fuse tracks provided by the local interacting multiple model filters. During the fusion process, the duplicate information is removed by considering the first order redundant information between the local tracks. With extensive simulations, we show that the proposed algorithm improves the tracking accuracy in ballistic target tracking in the re-entry phase applications.

## 1. Introduction

The problem of ballistic target tracking in the re-entry phase has attracted much attention due to both of its theoretical and practical significance. Technically speaking we need to develop a stochastic nonlinear filter for state estimation with respect to the ballistic target dynamics. Practical applications are in the fields of surveillance for safety against the re-entry of space debris produced by old satellites or spacecraft at the end of their lifetime [1,2,3]. Tracking a ballistic target has been identified as a stressful filtering problem due to the strong nonlinearity exhibited by the forces acting on the target [2,4,5]. Besides, the nonlinearity also results from the bearing and range measurements which are given with nonlinear measurement functions in relation to the target [6]. In the literature several nonlinear filtering methods have been applied to this tracking problem under the assumption that the ballistic coefficient is already known [7], including extended Kalman filter [8], unscented Kalman filter [9], ensemble Kalman filter [10], cubature Kalman filter [11] and particle filter [3]. The tracking problem becomes more difficult when the ballistic coefficient is known crudely or totally unknown. In such a case, an approach which has been investigated to be effective is the multiple model approach [12,13,14,15]. The structure of multiple model filter consists of a bank of local filters, with each filter modelled with a different value of ballistic coefficient. The output of the multiple model filter is in terms of Gaussian mixtures obtained by weighted mixing of the estimate from each local filter.

The technology of track-to-track fusion (T2TF) makes it possible to achieve further improvement of tracking accuracy than in the single sensor case. A basic T2TF system [16] consists of two sensors and a fusion center. Each sensor generates a local track by observing the target of interest and estimating with filtering. The fusion center fuses local tracks to obtain a fused track with improved accuracy. There has been a great deal of work in developing T2TF algorithms [17,18,19,20], however, most of these algorithms have been designed for systems with Gaussian density based filters (e.g., Kalman filter, extended Kalman filter and unscented Kalman filter) and may not be applicable to systems with multiple model filters. Demand for tracking ballistic targets with unknown ballistic coefficients poses a new requirement on using multiple model filters, which makes it necessary to fuse local tracks in terms of the Gaussian mixtures model [21] for tracking accuracy improvement. In the case of Gaussian mixtures, an empirical study into the use of Chernoff information has been investigated in [22], and an approximate approach for Chernoff fusion of Gaussian mixtures has been proposed in [23]. One drawback of this approach is that an exhaustive search must be performed to find the optimal weighting parameter [24], which makes it computationally intensive and difficult to implement in practical systems.

In this study, we consider a distributed ballistic target tracking scenario where the estimates of the local sensors are to be fused. We assume that the local sensors run IMM filters for handling unknown ballistic coefficient and the output of the local estimators are Gaussian mixtures. As a result, the track-to-track fusion problem we consider involves the fusion of Gaussian mixtures densities. The main contribution of this paper is to obtain the common information between the local estimates by considering the first order redundant information and derive the required fusion expressions to be employed in the fusion center. The proposed fusion rule is computationally efficient since it yields an analytical fused estimate for Gaussian mixtures. Simulation results show that the track-to-track fusion with Gaussian mixtures provides estimation accuracy improvement over the single Gaussian case. The remainder of the paper is organized as follows: the problem is formulated in Section 2. The Gaussian mixtures fusion rule is presented in Section 3. The simulation results are given in Section 4. The conclusions are presented in Section 5.

## 2. Problem Formulation

### 2.1. Target Dynamics

The kinematics of the ballistic target in the re-entry phase is derived under the following assumptions [7]: the forces acting on the target include Earth’s gravity and the aerodynamic drag; the effects of centrifugal acceleration, Coriolis acceleration, wind, lift force and spinning motion are ignored due to their minor effect on the target trajectory. Thus the system dynamics of a ballistic target [3,6] is given by:

(1) $$x_{k}=\Psi \left(x_{k-1}\right)+G\left[\begin{array}{c} 0\\ -g \end{array}\right]+w_{k}$$

where $x_{k-1}={\left[x_{p,k-1},x_{v,k-1},y_{p,k-1},y_{v,k-1}\right]}^{T}$ is the target state vector at time step k−1. $x_{p,k-1}$ and $y_{p,k-1}$ represent positions, $x_{v,k-1}$ and $y_{v,k-1}$ represent velocities in Cartesian coordinates (x,y). $w_{k}$ is a zero-mean white Gaussian process noise vector with covariance Q given by:

(2) $$Q=q\left[\begin{array}{cc} \Theta & 0\\ 0 & \Theta \end{array}\right]\text{ with }\Theta =\left[\begin{array}{cc} T^{3}/3 & T^{2}/2\\ T^{2}/2 & T \end{array}\right]$$

where q is a parameter related to the process noise intensity. The nonlinear function Ψ(·) in Equation (1) is given by:

(3) $$\Psi \left(x_{k-1}\right)=Fx_{k-1}+Gf\left(x_{k-1}\right)$$

where matrices F and G are given by:

(4) $$F=\left[\begin{array}{cccc} 1 & T & 0 & 0\\ 0 & 1 & 0 & 0\\ 0 & 0 & 1 & T\\ 0 & 0 & 0 & 1 \end{array}\right],\;G=\left[\begin{array}{cc} T^{2}/2 & 0\\ T & 0\\ 0 & T^{2}/2\\ 0 & T \end{array}\right]$$

and f(⋅) is the aerodynamic drag given by

(5) $$f\left(x_{k-1}\right)=-\frac{g\rho \left(y_{p,k-1}\right)\sqrt{x_{v,k-1}^{2}+y_{v,k-1}^{2}}\left[\begin{array}{c} x_{v,k-1}\\ y_{v,k-1} \end{array}\right]}{2\beta }$$

where β is the ballistic coefficient, g is the gravity acceleration, ρ(⋅) is the air density [25] which decays exponentially with the altitude $y_{p,k-1}$ as

(6) $$\rho \left(y_{p,k-1}\right)=c_{1}e^{-c_{2}y_{p,k-1}}$$

### 2.2. Sensor Configurations and Measurement Model

The sensor configuration involves two ground-radar sensors $S_{1}$ and $S_{2}$, which are located at $\left(x_{R}^{S_{l}},y_{R}^{S_{l}}\right)$ for l=1,2 respectively. The measurement equation is given by:

(7) $$z_{k}^{S_{l}}=h\left(x_{k}\right)+v_{k}^{S_{l}}\text{ for }l=1,2$$

where $z_{k}^{S_{l}}={\left[r_{k}^{S_{l}},\theta_{k}^{S_{l}}\right]}^{T}$ is the measurement vector, $v_{k}={\left[v_{r,k}^{S_{l}},v_{\theta ,k}^{S_{l}}\right]}^{T}$ is the measurement noise for sensor $S_{l}$. The sensor measures the range $r_{k}^{S_{l}}$ and the bearing $\theta_{k}$ in relation to the target. Thus the measurement model can be written as

(8) $$\begin{array}{l} r_{k}^{S_{l}}=\sqrt{{\left(x_{p,k}^{S_{l}}-x_{R}^{S_{l}}\right)}^{2}+{\left(y_{p,k}^{S_{l}}-y_{R}^{S_{l}}\right)}^{2}}+v_{r,k}^{S_{l}}\\ \theta_{k}^{S_{l}}=\tan^{-1}\left(\frac{y_{p,k}^{S_{l}}-y_{R}^{S_{l}}}{x_{p,k}^{S_{l}}-x_{R}^{S_{l}}}\right)+v_{\theta ,k}^{S_{l}} \end{array}\text{ for }l=1,2$$

where

(9) $$\begin{array}{c} v_{r,k}^{S_{l}}∼N\left(0,R_{r}^{S_{l}}\right)\\ v_{\theta ,k}^{S_{l}}∼N\left(0,R_{\theta }^{S_{l}}\right) \end{array}$$

### 2.3. Local Filter

Since the actual ballistic coefficient is unknown to the sensors, an interactive multiple model (IMM) filter [26] with r=2 models is applied at the single sensor level. The models are based on the extended Kalman filter (EKF), but having different value of ballistic coefficient $\beta_{i}^{S_{l}},i=1,\cdots ,r$. In general, the IMM filter merges state estimates computed under each possible model using local filters, with each filter using a different combination of the previous model-conditioned estimates [27]. The recursion equations for sensor $S_{l}$ consist of four major steps in each cycle as follows.

Step 1: Calculation of the mixing probabilities for i,j=1,⋯,r

(10) $$\mu_{i|j,k-1|k-1}^{S_{l}}=\frac{1}{{\bar{c}}_{j}^{S_{l}}}p_{ij}^{S_{l}}\mu_{i,k-1}^{S_{l}}$$

where ${\bar{c}}_{j}^{S_{l}}=\sum_{i=1}^{r}p_{ij}^{S_{l}}\mu_{i,k-1}^{S_{l}}$.

Step 2: Mixing for j=1,⋯,r:

(11) $$\begin{array}{l} {\hat{x}}_{0j,k-1|k-1}^{S_{l}}=\sum_{i=1}^{r}{\hat{x}}_{i,k-1|k-1}^{S_{l}}u_{i|j,k-1|k-1}^{S_{l}}\\ P_{0j,k-1|k-1}^{S_{l}}=\sum_{i=1}^{r}\mu_{i|j,k-1|k-1}^{S_{l}}\left[P_{i,k-1|k-1}^{S_{l}}+\left({\hat{x}}_{i,k-1|k-1}^{S_{l}}-{\hat{x}}_{0j,k-1|k-1}^{S_{l}}\right){\left({\hat{x}}_{i,k-1|k-1}^{S_{l}}-{\hat{x}}_{0j,k-1|k-1}^{S_{l}}\right)}^{T}\right] \end{array}$$

Step 3: Mode-matched filtering for j=1,⋯,r

(12) $$\begin{array}{l} {\hat{x}}_{j,k|k-1}^{S_{l}}=\Psi \left({\hat{x}}_{0j,k-1|k-1}^{S_{l}}\right)+G\left[\begin{array}{c} 0\\ -g \end{array}\right]\\ P_{j,k|k-1}^{S_{l}}=\left(F+Gf_{J,j,k}^{S_{l}}\right)P_{0j,k-1|k-1}^{S_{l}}{\left(F+Gf_{J,j,k}^{S_{l}}\right)}^{T}+Q \end{array}$$

(13) $$\begin{array}{l} {\hat{x}}_{j,k|k}^{S_{l}}={\hat{x}}_{j,k|k-1}^{S_{l}}+K_{k}^{S_{l}}\left(z_{k}^{S_{l}}-h\left({\hat{x}}_{j,k|k-1}^{S_{l}}\right)\right)\\ P_{j,k|k}^{S_{l}}=\left(I-K_{k}^{S_{l}}h_{J,j,k}^{S_{l}}\right)P_{j,k|k-1}^{S_{l}} \end{array}$$

where $K_{k}^{S_{l}}=P_{j,k|k-1}^{S_{l}}h_{J,j,k}^{T}{\left(h_{J,j,k}P_{j,k|k-1}^{S_{l}}h_{J,j,k}^{T}+R^{S_{l}}\right)}^{-1}$ and $R^{S_{l}}=\left[\begin{array}{cc} R_{r}^{S_{l}} & 0\\ 0 & R_{\theta ,k}^{S_{l}} \end{array}\right]$. $f_{J,j,k}^{S_{l}}$ is the Jacobian of $f^{S_{l}}\left(\cdot \right)$ calculated at the estimated state ${\hat{x}}_{0j,k-1|k-1}^{S_{l}}$, $h_{J,j,k}^{S_{l}}$ is the Jacobian of $h^{S_{l}}\left(\cdot \right)$ calculated at the estimated state ${\hat{x}}_{j,k|k-1}^{S_{l}}$. The detailed derivation of $f_{J,j,k}^{S_{l}}$ and $h_{J,j,k}^{S_{l}}$ for models given in Equations (5) and (8) are shown in Appendix A and Appendix B respectively.

Step 4: Mode probability update for j=1,⋯,r

(14) $$\begin{array}{lll} \Lambda_{j,k}^{S_{l}} & = & N\left(z_{k}^{S_{l}};h\left({\hat{x}}_{j,k|k-1}^{S_{l}}\right),h_{J,j,k}P_{j,k|k-1}^{S_{l}}h_{J,j,k}^{T}+R^{S_{l}}\right)\\ \mu_{j,k}^{S_{l}} & = & \frac{1}{c^{S_{l}}}\Lambda_{j,k}^{S_{l}}{\bar{c}}_{j}^{S_{l}}\\ c^{S_{l}} & = & \sum_{j=1}^{r}\Lambda_{j,k}^{S_{l}}{\bar{c}}_{j}^{S_{l}} \end{array}$$

**Figure 1:**
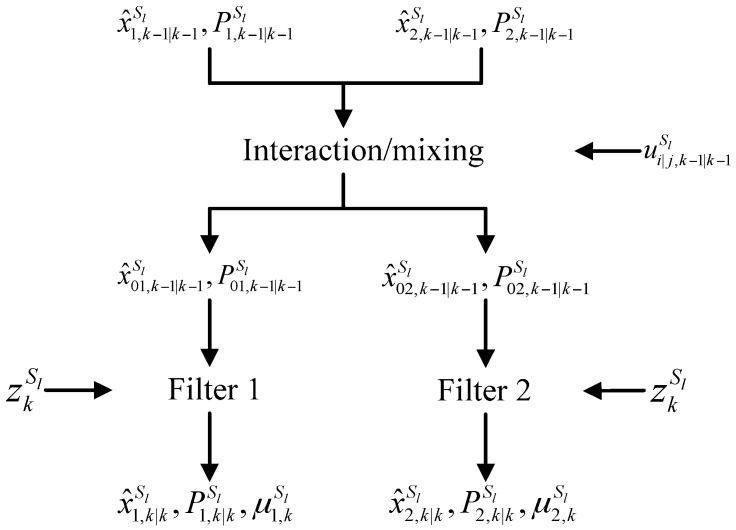
Structure of the IMM algorithm for sensor *S*_1_.

A block diagram for one cycle of the IMM filter with two models (r=2) for sensor $S_{l}$ is given in Figure 1.

### 2.4. Fusion Architecture

**Figure 2:**
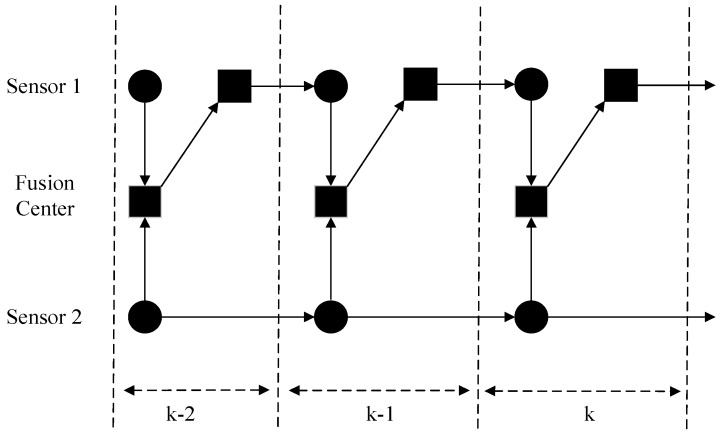
Fusion architecture.

Among the various choices for multiple model filters, the IMM filter has been shown to be one of the most effective schemes for hybrid systems [28]. In this study we assume that the local sensors run IMM filters for handling unknown ballistic coefficient, therefore the output of each local sensor is a Gaussian mixtures model. In this regard, the track-to-track fusion (T2TF) problem we consider here involves the fusion of Gaussian mixtures densities. As shown in Figure 2, the fusion architecture presented includes two ground-radar sensors *S*_1_ and *S*_2_ with local measurements obtained periodically and local tracks updated synchronously. In addition, the fusion center may transmit the latest fused track back to one local sensor. When the local sensor receives the fused track, it will be used to replace the local one. In practice, this condition usually happens when the fusion center is collocated with one local sensor [29]. In this study, we assume that after each fusion, the local track of sensor *S*_1_ is replaced with the fused track from the fusion center, while sensor *S*_2_ operates independently without information feedback from the fusion center.

## 3. Gaussian Mixtures Fusion

### 3.1. Basic Fusion Process and Redundant Information

The basic fusion process [19] is formulated as:

(15) $$p^{f}\left(x\right)=\frac{1}{c}\frac{p^{S_{1}}\left(x\right)p^{S_{2}}\left(x\right)}{p^{c}\left(x\right)}$$

where $p^{f}\left(x\right)$ is the fused estimate, $p^{S_{1}}\left(x\right)$ and $p^{S_{2}}\left(x\right)$ are the estimates from local sensors $S_{1}$ and $S_{2}$ respectively, c is a normalization constant. The common information between the local estimates is given in the denominator by $p^{c}\left(x\right)$, which is to be subtracted out. While the removal of duplicate information is straightforward in the theoretical formulation [30], identification of duplicate information for distributed estimation system can be difficult in practical implementation. In this paper, the first order redundant information [16,31] is considered. In this way, the fusion center only needs to keep track of the previous data received from sensor *S*_2_ at the previous communication time step and remove it when fusing the current estimates from sensor *S*_1_ and *S*_2_.

Specifically, when both $p^{S_{1}}\left(x\right)$ and $p^{S_{2}}\left(x\right)$ are Gaussian mixtures, namely:

(16) $$p^{S_{l}}\left(x\right)=\sum_{i=1}^{r}\mu_{i,k}^{S_{l}}N\left(x;{\hat{x}}_{i,k|k}^{S_{l}},P_{i,k|k}^{S_{l}}\right)\text{ for }l=1,2$$

where r=2 since the IMM filter is with two models for each sensor. Then the common information at time step k can be obtained as:

(17) $$p^{c}\left(x\right)=\sum_{i=1}^{2}\mu_{i,k}^{c}N\left(x;{\hat{x}}_{i,k|k}^{c},P_{i,k|k}^{c}\right)$$

where:

(18) $$\begin{array}{ccc} \mu_{i,k}^{c} & = & \sum_{j=1}^{r}\mu_{j,k-1}^{S_{2}}\mu_{j|i,k-1|k-1}^{S_{2}}\\ {\hat{x}}_{i,k|k}^{c} & = & \Psi \left({\hat{x}}_{0i,k-1|k-1}^{S_{2}}\right)+G\left[\begin{array}{c} 0\\ -g \end{array}\right]\\ P_{i,k|k}^{c} & = & \left(F+Gf_{J,i,k}^{S_{2}}\right)P_{0i,k-1|k-1}^{S_{2}}{\left(F+Gf_{J,i,k}^{S_{2}}\right)}^{T}+Q \end{array}\text{ for }i=1,2$$

and $f_{J,i,k}^{S_{2}}\left(\cdot \right)$ is the Jacobian calculated at the estimated state ${\hat{x}}_{0i,k-1|k-1}^{S_{2}}$.

### 3.2. Fusion of Gaussian Mixtures

In a distributed fusion problem, assuming two Gaussian mixtures, $p^{S_{1}}\left(x\right)=\sum_{i=1}^{2}\mu_{i,k}^{S_{1}}N\left(x;{\hat{x}}_{i,k|k}^{S_{1}},P_{i,k|k}^{S_{1}}\right)$ and $p^{S_{2}}\left(x\right)=\sum_{i=1}^{2}\mu_{i,k}^{S_{2}}N\left(x;{\hat{x}}_{i,k|k}^{S_{2}},P_{i,k|k}^{S_{2}}\right)$ are to be fused with a common prior distribution $p^{c}\left(x\right)$. With the standard Bayesian fusion formula, i.e., Equation (15), the fused probability density function (PDF) can be obtained as:

(19) $$p^{f}\left(x\right)=\frac{1}{c}\sum_{i=1}^{2}\sum_{j=1}^{2}\mu_{i,k}^{S_{1}}\mu_{j,k}^{S_{2}}\frac{N\left(x;{\hat{x}}_{i,k|k}^{S_{1}},P_{i,k|k}^{S_{1}}\right)N\left(x;{\hat{x}}_{j,k|k}^{S_{2}},P_{j,k|k}^{S_{2}}\right)}{p^{c}\left(x\right)}$$

To obtain an analytical fused result and avoid the potential complexity, one idea is to approximate the denominator $p^{c}\left(x\right)$ with a single Gaussian PDF using moment matching [27], namely:

(20) $$p^{c}\left(x\right)=\sum_{i=1}^{2}\mu_{i,k}^{c}N\left(x;{\hat{x}}_{i,k|k}^{c},P_{i,k|k}^{c}\right)\approx N\left(x;{\hat{x}}_{k|k}^{c},P_{k|k}^{c}\right)$$

where:

(21) $$\begin{array}{c} {\hat{x}}_{k|k}^{c}=\sum_{i=1}^{2}\mu_{i,k}^{c}{\hat{x}}_{i,k|k}^{c}\\ P_{k|k}^{c}=\sum_{i=1}^{2}\mu_{i,k}^{c}\left[P_{i,k|k}^{c}+\left({\hat{x}}_{i,k|k}^{c}-{\hat{x}}_{k|k}^{c}\right){\left({\hat{x}}_{i,k|k}^{c}-{\hat{x}}_{k|k}^{c}\right)}^{T}\right] \end{array}$$

With this approximation, the fusion expressions to be applied in IMM filter which is the major contribution of the current work are derived as follows:

(22) $$\begin{array}{ll} p^{f}\left(x\right) & \approx \frac{1}{c}\sum_{i=1}^{2}\sum_{j=1}^{2}\mu_{i,k}^{S_{1}}\mu_{j,k}^{S_{2}}\frac{N\left(x;{\hat{x}}_{i,k|k}^{S_{1}},P_{i,k|k}^{S_{1}}\right)N\left(x;{\hat{x}}_{j,k|k}^{S_{2}},P_{j,k|k}^{S_{2}}\right)}{N\left(x;{\hat{x}}_{k|k}^{c},P_{k|k}^{c}\right)}\\ & =\frac{1}{c}\sum_{i=1}^{2}\sum_{j=1}^{2}\mu_{i,k}^{S_{1}}\mu_{j,k}^{S_{2}}c_{ij,k|k}N\left(x;{\hat{x}}_{ij,k|k},P_{ij,k|k}\right) \end{array}$$

where

(23) $$\begin{array}{ccc} P_{ij,k|k}^{-1} & = & {\left(P_{i,k|k}^{S_{1}}\right)}^{-1}+{\left(P_{j,k|k}^{S_{2}}\right)}^{-1}-{\left(P_{k|k}^{c}\right)}^{-1}\\ P_{ij,k|k}^{-1}{\hat{x}}_{ij,k|k} & = & {\left(P_{i,k|k}^{S_{1}}\right)}^{-1}{\hat{x}}_{i,k|k}^{S_{1}}+{\left(P_{j,k|k}^{S_{2}}\right)}^{-1}{\hat{x}}_{j,k|k}^{S_{2}}-{\left(P_{k|k}^{c}\right)}^{-1}{\hat{x}}_{k|k}^{c} \end{array}$$

and

(24) $$c_{ij,k|k}=\exp\left(\zeta_{i,k|k}^{S_{1}}+\zeta_{j,k|k}^{S_{2}}-\zeta_{k|k}^{c}-\zeta_{ij,k|k}\right)$$

with

(25) $$\begin{array}{ccc} \zeta_{i,k|k}^{S_{1}} & = & -\frac{1}{2}\left(d\log2\pi -\log\left|{\left(P_{i,k|k}^{S_{1}}\right)}^{-1}\right|+{\left({\hat{x}}_{i,k|k}^{S_{1}}\right)}^{T}{\left(P_{i,k|k}^{S_{1}}\right)}^{-T}{\hat{x}}_{i,k|k}^{S_{1}}\right)\\ \zeta_{j,k|k}^{S_{2}} & = & -\frac{1}{2}\left(d\log2\pi -\log\left|{\left(P_{j,k|k}^{S_{2}}\right)}^{-1}\right|+{\left({\hat{x}}_{j,k|k}^{S_{2}}\right)}^{T}{\left(P_{j,k|k}^{S_{2}}\right)}^{-T}{\hat{x}}_{j,k|k}^{S_{2}}\right)\\ \zeta_{k|k}^{c} & = & -\frac{1}{2}\left(d\log2\pi -\log\left|{\left(P_{k|k}^{c}\right)}^{-1}\right|+{\left({\hat{x}}_{k|k}^{c}\right)}^{T}{\left(P_{k|k}^{c}\right)}^{-T}{\hat{x}}_{k|k}^{c}\right)\\ \zeta_{ij,k|k} & = & -\frac{1}{2}\left(d\log2\pi -\log\left|P_{ij,k|k}^{-1}\right|+{\hat{x}}_{ij,k|k}^{T}P_{ij,k|k}^{-T}{\hat{x}}_{ij,k|k}\right) \end{array}$$

The derivation of Equations (23)–(25) is presented in Appendix C.

### 3.3. Gaussian Mixtures Reduction

As one can see from Equation (22), the fused Gaussian mixtures PDF has an exponentially growing number of components as more Gaussian mixtures are multiplied in the long run. Thus it is necessary to manage the components growth with a method of Gaussian mixtures reduction. Suppose that we are given a Gaussian mixtures model with n components, and we wish to approximate it with a mixture of r components (r<n). In general, the Gaussian mixtures reduction algorithm can be operated in the following manner.

While more than r components remain, choose two components that in a sense to be least dissimilar and replace them by their moment matching merge as:

(26) $$w_{m}=w_{i}+w_{j}$$

(27) $${\hat{x}}_{m}=\frac{w_{i}}{w_{m}}{\hat{x}}_{i}+\frac{w_{j}}{w_{m}}{\hat{x}}_{j}$$

(28) $$P_{m}=\sum_{i=1}^{2}\frac{w_{i}}{w_{m}}\left[P_{i}+\left({\hat{x}}_{i}-{\hat{x}}_{m}\right){\left({\hat{x}}_{i}-{\hat{x}}_{m}\right)}^{T}\right]$$

where $\left(w_{i},{\hat{x}}_{i},P_{i}\right)$ and $\left(w_{j},{\hat{x}}_{j},P_{j}\right)$ are two weighted Gaussian components to be merged, and $\left(w_{m},{\hat{x}}_{m},P_{m}\right)$ is their moment matching approximation.

For the dissimilarity measure between two components of a Gaussian mixtures model, we adopt a metric that is proposed in Reference [32] as an upper bound on the discrimination of the Gaussian mixtures after the merge from the Gaussian mixtures before the merge. The dissimilarity measure is given as follows:

(29) $$D\left(\left(w_{i},{\hat{x}}_{i},P_{i}\right),\left(w_{j},{\hat{x}}_{j},P_{j}\right)\right)≜\frac{\left(w_{i}+w_{j}\right)\ln\left(\det\left(P_{m}\right)\right)-w_{i}\ln\left(\det\left(P_{i}\right)\right)-w_{j}\ln\left(\det\left(P_{j}\right)\right)}{2}$$

Thus in each iteration, a Gaussian mixtures model with n components is constructed after the Gaussian mixtures fusion, then we operate the procedure of Gaussian mixtures reduction in an iterative manner until n=r, so as to meet the requirement that the IMM filter is set to be with r models. In our case, the fused Gaussian mixtures consist of n=4 components after fusion in Equation (22), namely:

(30) $$p^{f}\left(x\right)=\sum_{i=1}^{2}\sum_{j=1}^{2}w_{ij}N\left(x;{\hat{x}}_{ij},P_{ij}\right)$$

where:

(31) $$w_{ij}=\frac{\mu_{i}^{S_{1}}\mu_{j}^{S_{2}}c_{ij}}{c}$$

**Figure 3:**
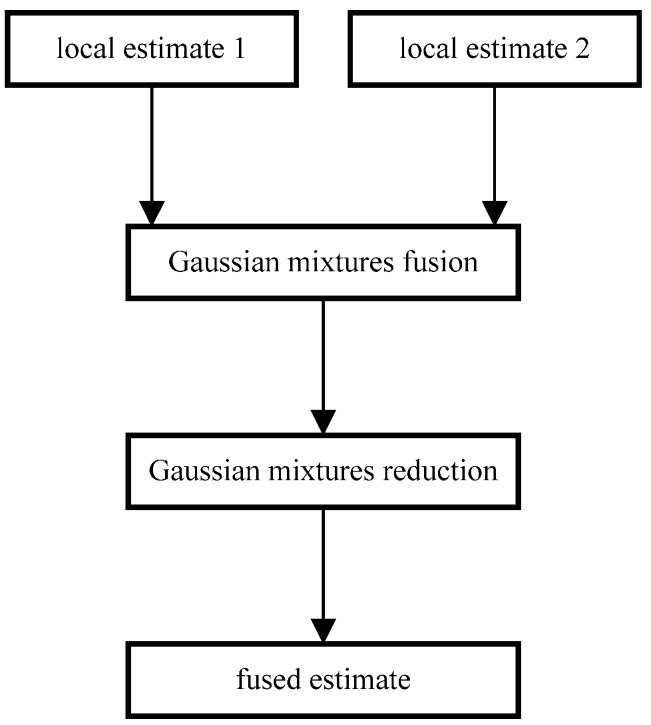
Flow chart of the fusion methodology.

Note that the subscript k|k is omitted for the sake of brevity. We set $\left(w_{11},{\hat{x}}_{11},P_{11}\right)$ and $\left(w_{22},{\hat{x}}_{22},P_{22}\right)$ as major components, since they are consistent with model 1 and 2 in a straightforward manner. For the rest two cross-components, namely $\left(w_{12},{\hat{x}}_{12},P_{12}\right)$ and $\left(w_{21},{\hat{x}}_{21},P_{21}\right)$, they are supposed to be merged with one of the major components according to the dissimilarity measure given in Equation (29). More specifically, for $\left(w_{12},{\hat{x}}_{12},P_{12}\right)$, if $D\left(\left(w_{12},{\hat{x}}_{12},P_{12}\right),\left(w_{11},{\hat{x}}_{11},P_{11}\right)\right)<D\left(\left(w_{12},{\hat{x}}_{12},P_{12}\right),\left(w_{22},{\hat{x}}_{22},P_{22}\right)\right)$, then we merge $\left(w_{12},{\hat{x}}_{12},P_{12}\right)$ with $\left(w_{11},{\hat{x}}_{11},P_{11}\right)$, otherwise we merge $\left(w_{12},{\hat{x}}_{12},P_{12}\right)$ with $\left(w_{22},{\hat{x}}_{22},P_{22}\right)$; for $\left(w_{21},{\hat{x}}_{21},P_{21}\right)$, if $D\left(\left(w_{21},{\hat{x}}_{21},P_{21}\right),\left(w_{11},{\hat{x}}_{11},P_{11}\right)\right)<D\left(\left(w_{21},{\hat{x}}_{21},P_{21}\right),\left(w_{22},{\hat{x}}_{22},P_{22}\right)\right)$, then we merge $\left(w_{21},{\hat{x}}_{21},P_{21}\right)$ with $\left(w_{11},{\hat{x}}_{11},P_{11}\right)$, otherwise we merge $\left(w_{21},{\hat{x}}_{21},P_{21}\right)$ with $\left(w_{22},{\hat{x}}_{22},P_{22}\right)$. In the end, a flow chart of the proposed fusion methodology is given in Figure 3 as follows:

## 4. Simulation Results

The proposed algorithm is applied in a re-entry ballistic target tracking scenario to verify the performance through a series of simulation runs. To the best author’s knowledge, the Gaussian mixtures fusion methodology has not been applied in a distributed ballistic target tracking scenario for track-to-track fusion before. The system dynamic model is represented as Equation (1), where we use *T* = 2 s, *q* = 1 m^2^·s^−3^ and *g* = 9.81 m·s^2^. The initial state $x_{0}$ is:

(32) $$x_{0}=\left[\begin{array}{c} 23200m\\ 2290\cos\left(190^{{}^{\circ}}\right)m\cdot s^{-1}\\ 88000m\\ 2290\sin\left(190^{{}^{\circ}}\right)m\cdot s^{-1} \end{array}\right]$$

with initial covariance:

(33) $$P_{0}=\left[\begin{array}{cccc} 1000^{2}m^{2} & 0 & 0 & 0\\ 0 & 20^{2}m^{2}\cdot s^{-2} & 0 & 0\\ 0 & 0 & 1000^{2}m^{2} & 0\\ 0 & 0 & 0 & 20^{2}m^{2}\cdot s^{-2} \end{array}\right]$$

For the air density ρ(⋅), we have *c*_1_ = 1.227, *c*_2_ = 1.093 × 10^−4^ for *y_p_* < 9144 m and, and *c*_1_ = 1.754, *c*_2_ = 1.49 × 10^−4^ for *y_p_* > 9144 m. The actual target ballistic coefficient is *β* = 40,000 kg·m^−1^·s^−2^.

**Figure 4:**
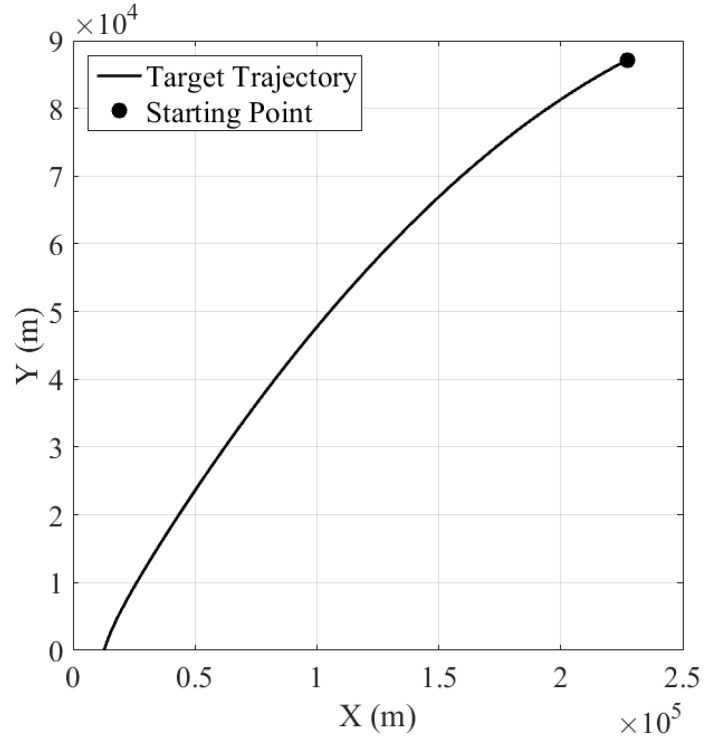
Ballistic target trajectory.

**Figure 5:**
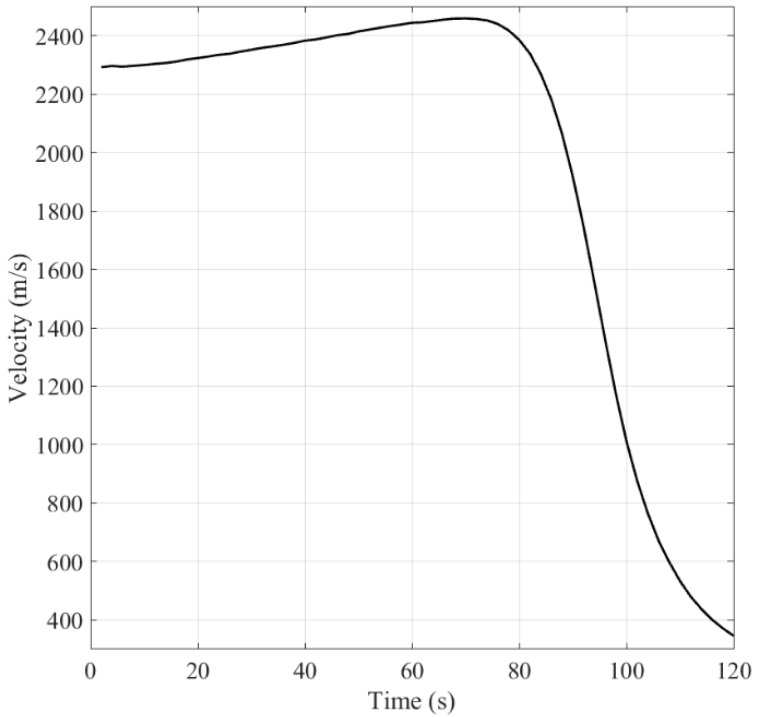
Velocity of ballistic target against time.

**Figure 6:**
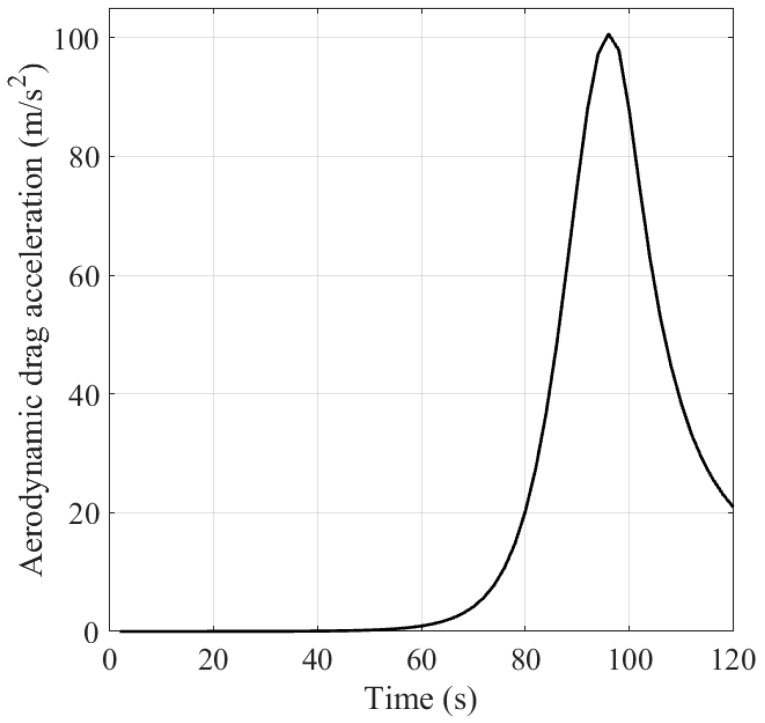
Aerodynamic drag acceleration against time.

Figure 4 shows the target trajectory in the X-Y plane. Figure 5 and Figure 6 show the velocity of the ballistic target, and the aerodynamic drag acceleration against time, respectively. It can be observed that the velocity decreases with the increment of aerodynamic drag f(⋅).

For the measurement model, we assume that the two sensors are homogeneous, in the sense that $R_{r}^{S_{1}}=R_{r}^{S_{2}}=100^{2}\;m^{2}$ and $R_{\theta }^{S_{1}}=R_{\theta }^{S_{2}}=0.05^{2}\;{\text{rad}}^{2}$. Besides, we consider that the sensors are located at $\left(x_{R}^{S_{1}},y_{R}^{S_{1}}\right)=\left(0,0\right)$ and $\left(x_{R}^{S_{2}},y_{R}^{S_{2}}\right)=\left(50,000,0\right)$, respectively.

For the local filters, it is assumed that the actual ballistic coefficient of the target is unknown to both sensors, so that $\beta_{1}^{S_{1}}=\beta_{1}^{S_{2}}=60,000\text{ kg}\cdot m^{-1}\cdot s^{-2}$ and $\beta_{2}^{S_{1}}=\beta_{2}^{S_{2}}=10,000\text{ kg}\cdot m^{-1}\cdot s^{-2}$ are used as the IMM models for both sensor $S_{1}$ and $S_{2}$. Besides, the Markov chain transition matrix was taken as:

(34) $$\left[p_{ij}\right]=\left[\begin{array}{cc} 0.95 & 0.05\\ 0.05 & 0.95 \end{array}\right]$$

The proposed Gaussian mixtures fusion method is verified by comparing the results between sensors *S*_1_ and *S*_2_. Note that the estimate from sensor *S*_1_ is identical to the fused result, for the reason that after each fusion the local track from sensor *S*_1_ is replaced with the fused track from the fusion center. Unlike sensor *S*_1_, the estimate from sensor *S*_2_ is solely obtained from one singe IMM filter, since sensor *S*_2_ operates by itself without information feedback from the fusion center.

**Figure 7:**
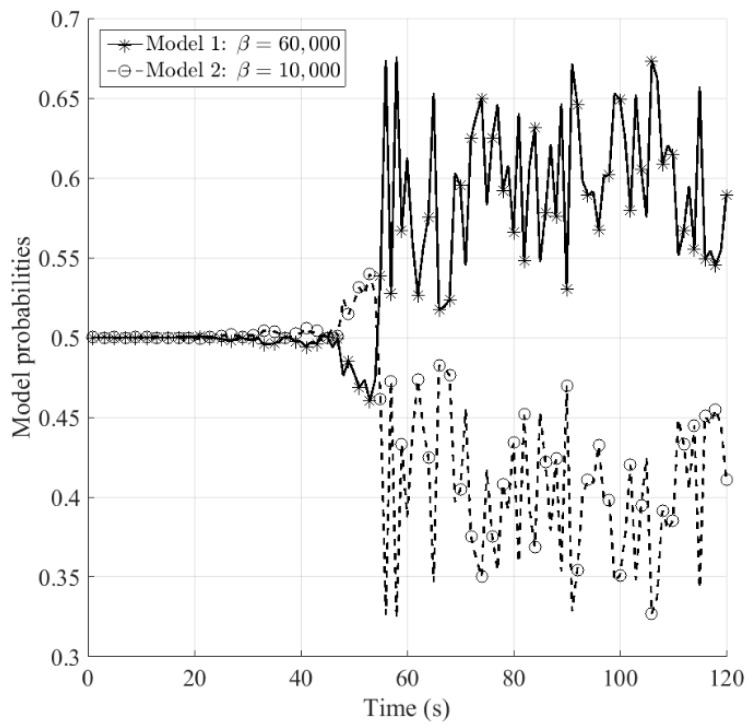
Model probabilities for sensor *S*_1_.

**Figure 8:**
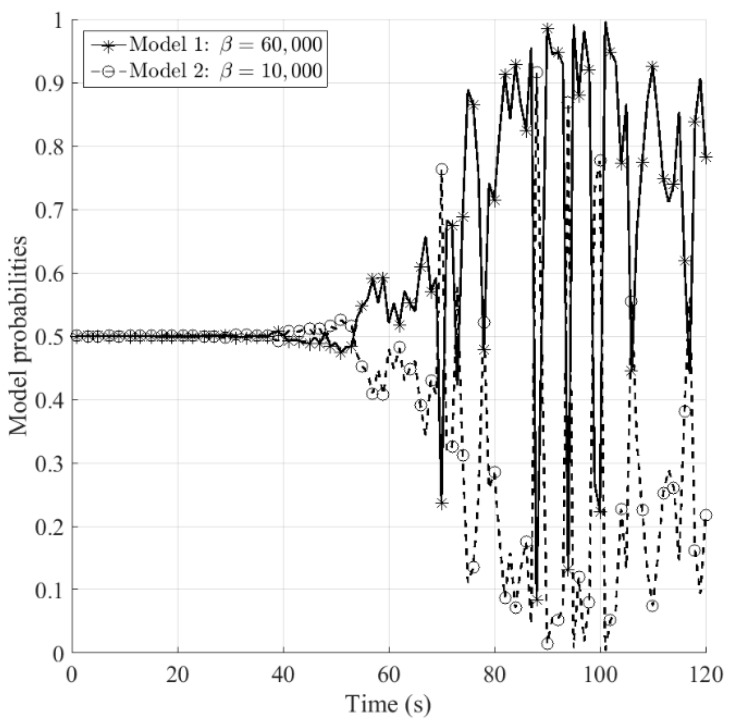
Model probabilities for sensor *S*_2_.

Figure 7 and Figure 8 show the estimated model probabilities for sensors *S*_1_ and *S*_2_, respectively. It can be seen that during 0–50 s the probabilities for models 1 and 2 are equal to each other, for the reason that the aerodynamic drag is zero which makes it not able to judge which model matches better. The ballistic coefficient becomes observable from *t* = 50 s, at that time the aerodynamic drag begins to emerge. It can be observed that the model probabilities for sensor *S*_1_ is more stable than that for sensor *S*_2_, namely the proposed fusion method improves the stability of the estimated model probabilities.

**Figure 9:**
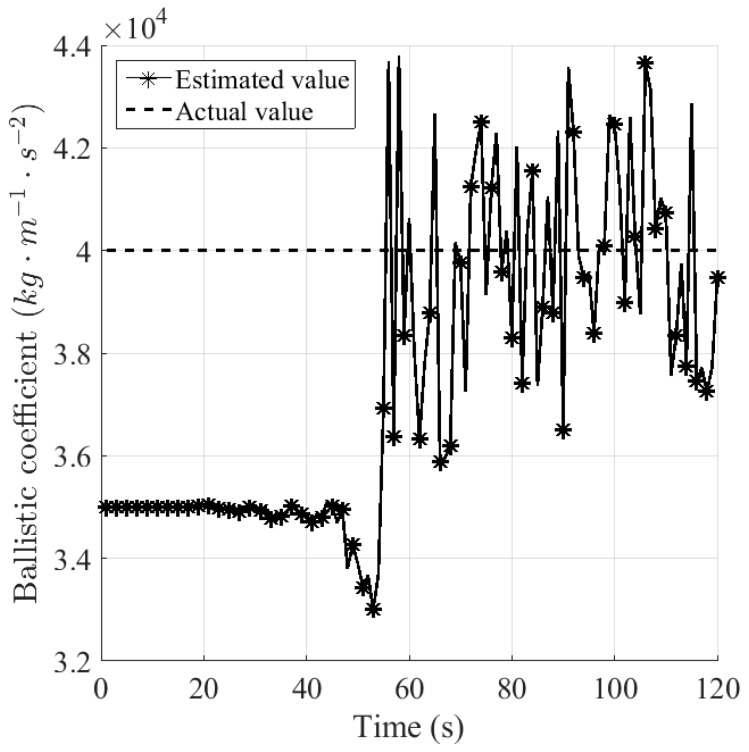
Estimated value of the ballistic coefficient for sensor *S*_1_.

**Figure 10:**
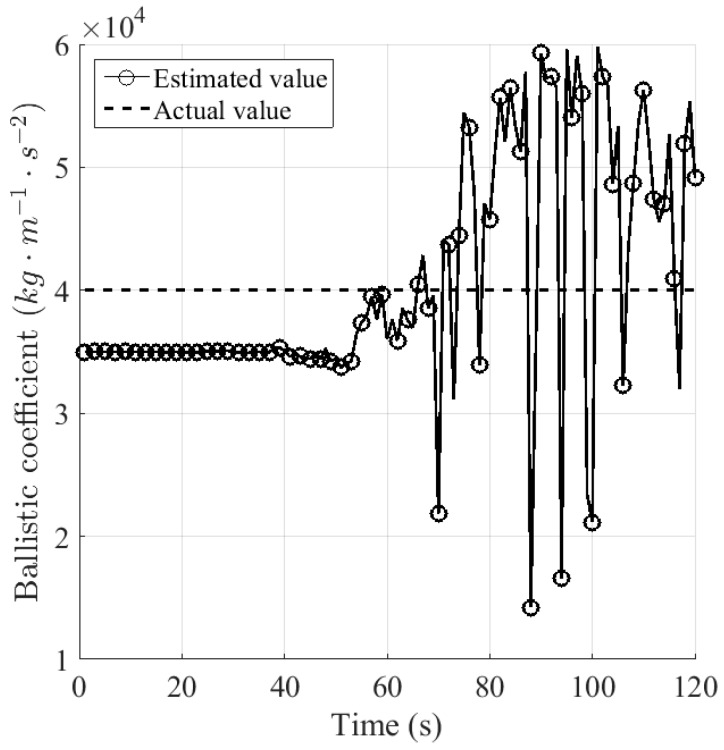
Estimated value of the ballistic coefficient for sensor *S*_2_.

We further calculate the estimated ballistic coefficient with the total probability theorem [25] as:

(35) $${\hat{\beta }}^{S_{l}}=\sum_{i=1}^{2}\beta_{i}^{S_{l}}\mu_{i}^{S_{l}}\text{ for }l=1,2$$

where $\mu_{i}^{S_{l}}$ is the estimated model probability for $\beta_{i}^{S_{l}}$. Figure 9 and Figure 10 plot the estimated value of ballistic coefficient from sensors *S*_1_ and *S*_2_, respectively. It can be seen that after the drag force emerges, the estimated value of the ballistic coefficient for sensor *S*_1F_, namely the fused one, appears to be more accurate and stable than that for sensor *S*_2_, in the sense that the first one ranges around [3.6,4.4] × 10^4^, and the latter one ranges around [1.5,6.0] × 10^4^.

**Figure 11:**
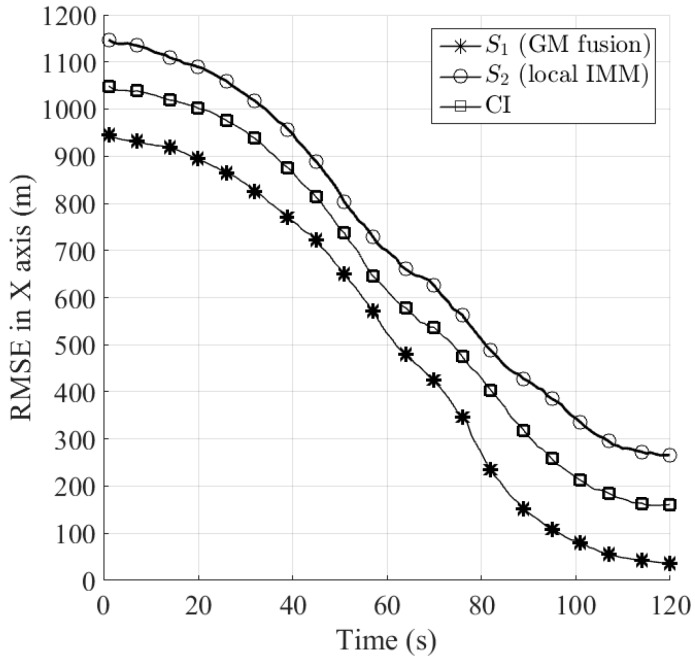
Comparison of RMSE in X axis between the proposed Gaussian mixtures (GM) fusion, local IMM and CI.

**Figure 12:**
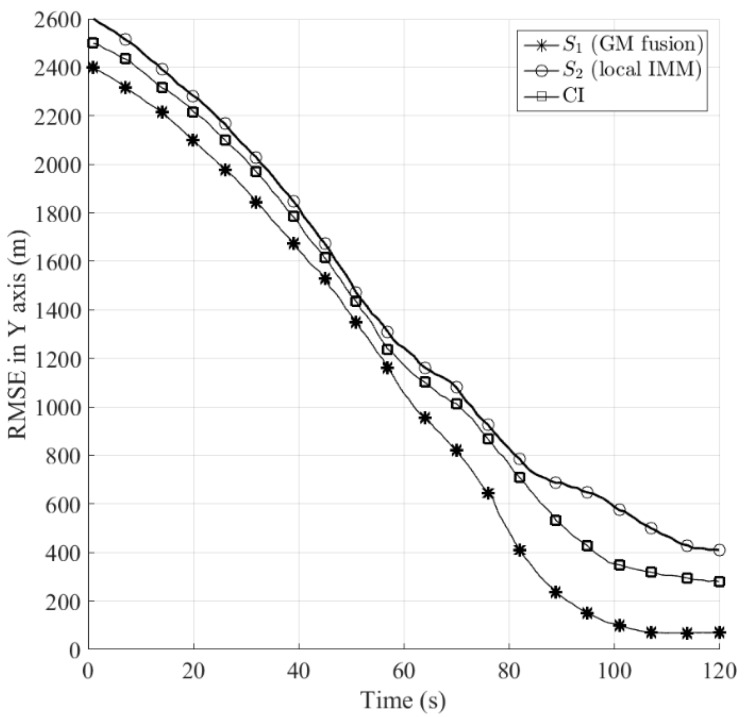
Comparison of RMSE in Y axis between the proposed Gaussian mixtures (GM) fusion, local IMM and CI.

In the following, we compare the position estimation accuracy in terms of root mean square error (RMSE) between the proposed Gaussian mixtures fusion method, the single IMM filter and the covariance intersection (CI) method. The CI method [18] is the most well-known fusion technique which yields consistent estimates by optimizing a nonlinear cost function associated with the fused covariance. For the reason that CI cannot be applied to Gaussian mixtures fusion directly, it requires one to approximate the output of IMM filter with the single Gaussian density using moment matching before track fusion. Figure 11 and Figure 12 and Table 1 show the RMSE of the algorithms in X and Y axes, respectively, by performing 1000 runs of Monte Carlo simulation. It can be seen that both of the fusion methods, namely the proposed Gaussian mixtures fusion, and CI provide performance improvement over the single IMM filter. Furthermore, the performance of the proposed Gaussian mixtures fusion is better than CI. The first reason is that we remove the first order redundant information between the local tracks during Gaussian mixtures fusion process, however, the CI only provides a conservative estimate due to its ignorance of the cross correlations between the local tracks.

The second reason is that instead of fusing Gaussian mixtures directly as for the proposed fusion method, for CI one needs to approximate the original local Gaussian mixtures estimate with mean and covariance before track fusion, thus the resulting approximation error could degrade the estimation performance.

## 5. Conclusions

A Gaussian mixtures fusion algorithm for the track-to-track fusion problem is proposed in this study. The common information to be reduced during the fusion process is approximated with the first order redundant information between the local tracks. The proposed fusion algorithm is applied to tracking a ballistic target with unknown ballistic coefficient using IMM filters. A series of Monte Carlo simulations are conducted to evaluate the sensor fusion performance. The results indicate that the proposed algorithm improves the estimation accuracy in terms of the root mean square error. There are two issues to be addressed in the future. First, a more detailed analysis of the effects of the first order approximation must be carried out. Second, experiments should be conducted to explore the effect of using higher order approximation to formulate the common information between the local estimates.

## Figures and Tables

**Table 1:** Comparison of RMSE (m) in X and Y axis (64 s–120 s).

*S*_1_		*S*_2_		CI	
X	Y	X	Y	X	Y
478.9	953.8	660.3	1163	578.2	1102
425.3	820.7	625.8	1081	537.1	1014
345.4	644.8	563.2	927.4	474.9	868.6
233.9	410	488.8	786.9	404.2	710.1
151.9	235.1	427.7	687	319	531.9
109.1	150.3	385.2	647.5	258.2	426.8
80.25	100.3	336.2	574.2	212.8	349
56.48	71.63	295.6	500.6	185	319.1
42.16	67.96	273.1	429.3	163.9	294.3
35.5	66.76	266.1	410.4	160.7	280.4

## Appendix A

(A1) $$f_{J,j,k}^{S_{l}}\left(1,1\right)=0$$

(A2) $$f_{J,j,k}^{S_{l}}\left(1,2\right)=-\frac{g\rho \left({\hat{x}}_{0j,k-1|k-1}^{S_{l}}\left(3\right)\right)}{2\beta^{S_{l}}}\cdot \frac{2{\left({\hat{x}}_{0j,k-1|k-1}^{S_{l}}\left(2\right)\right)}^{2}+{\left({\hat{x}}_{0j,k-1|k-1}^{S_{l}}\left(4\right)\right)}^{2}}{\sqrt{{\left({\hat{x}}_{0j,k-1|k-1}^{S_{l}}\left(2\right)\right)}^{2}+{\left({\hat{x}}_{0j,k-1|k-1}^{S_{l}}\left(4\right)\right)}^{2}}}$$

(A3) $$f_{J,j,k}^{S_{l}}\left(1,3\right)=\frac{gc_{2}\rho \left({\hat{x}}_{0j,k-1|k-1}^{S_{l}}\left(3\right)\right)}{2\beta^{S_{l}}}\cdot {\hat{x}}_{0j,k-1|k-1}^{S_{l}}\left(2\right)\sqrt{{\left({\hat{x}}_{0j,k-1|k-1}^{S_{l}}\left(2\right)\right)}^{2}+{\left({\hat{x}}_{0j,k-1|k-1}^{S_{l}}\left(4\right)\right)}^{2}}$$

(A4) $$f_{J,j,k}^{S_{l}}\left(1,4\right)=-\frac{g\rho \left({\hat{x}}_{0j,k-1|k-1}^{S_{l}}\left(3\right)\right)}{2\beta^{S_{l}}}\cdot \frac{\left({\hat{x}}_{0j,k-1|k-1}^{S_{l}}\left(2\right)\right)\left({\hat{x}}_{0j,k-1|k-1}^{S_{l}}\left(4\right)\right)}{\sqrt{{\left({\hat{x}}_{0j,k-1|k-1}^{S_{l}}\left(2\right)\right)}^{2}+{\left({\hat{x}}_{0j,k-1|k-1}^{S_{l}}\left(4\right)\right)}^{2}}}$$

(A5) $$f_{J,j,k}^{S_{l}}\left(2,1\right)=0$$

(A6) $$f_{J,j,k}^{S_{l}}\left(2,2\right)=-\frac{g\rho \left(\left({\hat{x}}_{0j,k-1|k-1}^{S_{l}}\left(3\right)\right)\right)}{2\beta^{S_{l}}}\cdot \frac{\left({\hat{x}}_{0j,k-1|k-1}^{S_{l}}\left(2\right)\right)\left({\hat{x}}_{0j,k-1|k-1}^{S_{l}}\left(4\right)\right)}{\sqrt{{\left({\hat{x}}_{0j,k-1|k-1}^{S_{l}}\left(2\right)\right)}^{2}+{\left({\hat{x}}_{0j,k-1|k-1}^{S_{l}}\left(4\right)\right)}^{2}}}$$

(A7) $$f_{J,j,k}^{S_{l}}\left(2,3\right)=\frac{gc_{2}\rho \left({\hat{x}}_{0j,k-1|k-1}^{S_{l}}\left(3\right)\right)}{2\beta^{S_{l}}}\times \left({\hat{x}}_{0j,k-1|k-1}^{S_{l}}\left(4\right)\right)\sqrt{{\left({\hat{x}}_{0j,k-1|k-1}^{S_{l}}\left(2\right)\right)}^{2}+{\left({\hat{x}}_{0j,k-1|k-1}^{S_{l}}\left(4\right)\right)}^{2}}$$

(A8) $$f_{J,j,k}^{S_{l}}\left(2,4\right)=\frac{g\;\rho \left({\hat{x}}_{0j,k-1|k-1}^{S_{l}}\left(3\right)\right)}{2\beta^{S_{l}}}\cdot \frac{{\hat{x}}_{0j,k-1|k-1}^{S_{l}}\left(2\right)+2{\hat{x}}_{0j,k-1|k-1}^{S_{l}}\left(4\right)}{\sqrt{{\left({\hat{x}}_{0j,k-1|k-1}^{S_{l}}\left(2\right)\right)}^{2}+{\left({\hat{x}}_{0j,k-1|k-1}^{S_{l}}\left(4\right)\right)}^{2}}}$$

## Appendix B

(B1) $$h_{J,j,k}^{S_{l}}\left(1,1\right)=\frac{{\hat{x}}_{j,k|k-1}^{S_{l}}\left(1\right)-x_{R}^{S_{l}}}{\sqrt{{\left({\hat{x}}_{j,k|k-1}^{S_{l}}\left(1\right)-x_{R}^{S_{l}}\right)}^{2}+{\left({\hat{x}}_{j,k|k-1}^{S_{l}}\left(3\right)-y_{R}^{S_{l}}\right)}^{2}}}$$

(B2) $$h_{J,j,k}^{S_{l}}\left(1,2\right)=0$$

(B3) $$h_{J,j,k}^{S_{l}}\left(1,3\right)=\frac{{\hat{x}}_{j,k|k-1}^{S_{l}}\left(3\right)-y_{R}^{S_{l}}}{\sqrt{{\left({\hat{x}}_{j,k|k-1}^{S_{l}}\left(1\right)-x_{R}^{S_{l}}\right)}^{2}+{\left({\hat{x}}_{j,k|k-1}^{S_{l}}\left(3\right)-y_{R}^{S_{l}}\right)}^{2}}}$$

(B4) $$h_{J,j,k}^{S_{l}}\left(1,4\right)=0$$

(B5) $$h_{J,j,k}^{S_{l}}\left(2,1\right)=-\frac{{\hat{x}}_{j,k|k-1}^{S_{l}}\left(3\right)-y_{R}^{S_{l}}}{{\left({\hat{x}}_{j,k|k-1}^{S_{l}}\left(1\right)-x_{R}^{S_{l}}\right)}^{2}+{\left({\hat{x}}_{j,k|k-1}^{S_{l}}\left(3\right)-y_{R}^{S_{l}}\right)}^{2}}$$

(B6) $$h_{J,j,k}^{S_{l}}\left(2,2\right)=0$$

(B7) $$h_{J,j,k}^{S_{l}}\left(2,3\right)=\frac{{\hat{x}}_{j,k|k-1}^{S_{l}}\left(1\right)-x_{R}^{S_{l}}}{{\left({\hat{x}}_{j,k|k-1}^{S_{l}}\left(1\right)-x_{R}^{S_{l}}\right)}^{2}+{\left({\hat{x}}_{j,k|k-1}^{S_{l}}\left(3\right)-y_{R}^{S_{l}}\right)}^{2}}$$

(B8) $$h_{J,j,k}^{S_{l}}\left(2,4\right)=0$$

## Appendix C

The multivariate Gaussian PDF $N\left(x;\hat{x},P\right)$ can be written in canonical notation [33] as:

(C1) $$\begin{array}{ll} p\left(x\right) & =N\left(x;\hat{x},P\right)\\ & =\exp\left(\zeta +y^{T}x-\frac{1}{2}x^{T}Yx\right) \end{array}$$

where:

(C2) $$\begin{array}{ccc} Y & = & P^{-1}\\ y & = & P^{-1}\hat{x}\\ \zeta & = & -\frac{1}{2}\left(d\log2\pi -\log\left|Y\right|+y^{T}Y^{-1}y\right) \end{array}$$

d is the dimensionality of x, |Y|≜det(Y).

Define:

(C3) $$\begin{array}{cc} N\left(x;{\hat{x}}_{i,k|k}^{S_{1}},P_{i,k|k}^{S_{1}}\right) & =\exp\left(\zeta_{i,k|k}^{S_{1}}+{\left(y_{i,k|k}^{S_{1}}\right)}^{T}x-\frac{1}{2}x^{T}Y_{i,k|k}^{S_{1}}x\right)\\ N\left(x;{\hat{x}}_{j,k|k}^{S_{2}},P_{j,k|k}^{S_{2}}\right) & =\exp\left(\zeta_{j,k|k}^{S_{2}}+{\left(y_{j,k|k}^{S_{2}}\right)}^{T}x-\frac{1}{2}x^{T}Y_{j,k|k}^{S_{2}}x\right)\\ N\left(x;{\hat{x}}_{k|k}^{c},P_{k|k}^{c}\right) & =\exp\left(\zeta_{k|k}^{c}+{\left(y_{k|k}^{c}\right)}^{T}x-\frac{1}{2}x^{T}Y_{k|k}^{c}x\right) \end{array}$$

so we have:

(C4) $$\begin{array}{l} \frac{N\left(x;{\hat{x}}_{i,k|k}^{S_{1}},P_{i,k|k}^{S_{1}}\right)N\left(x;{\hat{x}}_{j,k|k}^{S_{2}},P_{j,k|k}^{S_{2}}\right)}{N\left(x;{\hat{x}}_{k|k}^{c},P_{k|k}^{c}\right)}\\ =\exp\left[\left(\zeta_{i,k|k}^{S_{1}}+\zeta_{j,k|k}^{S_{2}}-\zeta_{k|k}^{c}\right)+{\left(y_{i,k|k}^{S_{1}}+y_{j,k|k}^{S_{2}}-y_{k|k}^{c}\right)}^{T}x-\frac{1}{2}x^{T}\left(Y_{i,k|k}^{S_{1}}+Y_{j,k|k}^{S_{2}}-Y_{k|k}^{c}\right)x\right] \end{array}$$

We further define:

(C5) $$\begin{array}{l} Y_{ij,k|k}≜Y_{i,k|k}^{S_{1}}+Y_{j,k|k}^{S_{2}}-Y_{k|k}^{c}\\ y_{ij,k|k}≜y_{i,k|k}^{S_{1}}+y_{j,k|k}^{S_{2}}-y_{k|k}^{c}\\ \zeta_{ij,k|k}≜-\frac{1}{2}\left(d\log2\pi -\log\left|Y_{ij,k|k}\right|+y_{ij,k|k}^{T}Y_{ij,k|k}^{-1}y_{ij,k|k}\right) \end{array}$$

Then we have:

(C6) $$\begin{array}{l} \frac{N\left(x;{\hat{x}}_{i,k|k}^{S_{1}},P_{i,k|k}^{S_{1}}\right)N\left(x;{\hat{x}}_{j,k|k}^{S_{2}},P_{j,k|k}^{S_{2}}\right)}{N\left(x;{\hat{x}}_{k|k}^{c},P_{k|k}^{c}\right)}\\ =\exp\left[\left(\zeta_{i,k|k}^{S_{1}}+\zeta_{j,k|k}^{S_{2}}-\zeta_{k|k}^{c}\right)-\zeta_{ij,k|k}+\zeta_{ij,k|k}+{\left(y_{i,k|k}^{S_{1}}+y_{j,k|k}^{S_{2}}-y_{k|k}^{c}\right)}^{T}x-\frac{1}{2}x^{T}\left(Y_{i,k|k}^{S_{1}}+Y_{j,k|k}^{S_{2}}-Y_{k|k}^{c}\right)x\right]\\ =\exp\left[\left(\zeta_{i,k|k}^{S_{1}}+\zeta_{j,k|k}^{S_{2}}-\zeta_{k|k}^{c}\right)-\zeta_{ij,k|k}+\zeta_{ij,k|k}+y_{ij,k|k}^{T}x-\frac{1}{2}x^{T}Y_{ij,k|k}x\right]\\ =\exp\left(\zeta_{i,k|k}^{S_{1}}+\zeta_{j,k|k}^{S_{2}}-\zeta_{k|k}^{c}-\zeta_{ij,k|k}\right)\cdot \exp\left(\zeta_{ij,k|k}+y_{ij,k|k}^{T}x-\frac{1}{2}x^{T}Y_{ij,k|k}x\right) \end{array}$$

Therefore $\frac{N\left(x;{\hat{x}}_{i,k|k}^{S_{1}},P_{i,k|k}^{S_{1}}\right)N\left(x;{\hat{x}}_{j,k|k}^{S_{2}},P_{j,k|k}^{S_{2}}\right)}{N\left(x;{\hat{x}}_{k|k}^{c},P_{k|k}^{c}\right)}$ is a scaled Gaussian PDF as

(C7) $$\frac{N\left(x;{\hat{x}}_{i,k|k}^{S_{1}},P_{i,k|k}^{S_{1}}\right)N\left(x;{\hat{x}}_{j,k|k}^{S_{2}},P_{j,k|k}^{S_{2}}\right)}{N\left(x;{\hat{x}}_{k|k}^{c},P_{k|k}^{c}\right)}=c_{ij,k|k}N\left(x;{\hat{x}}_{ij,k|k},P_{ij,k|k}\right)$$

where:

(C8) $$\begin{array}{ccc} P_{ij,k|k}^{-1} & = & {\left(P_{i,k|k}^{S_{1}}\right)}^{-1}+{\left(P_{j,k|k}^{S_{2}}\right)}^{-1}-{\left(P_{k|k}^{c}\right)}^{-1}\\ P_{ij,k|k}^{-1}{\hat{x}}_{ij,k|k} & = & {\left(P_{i,k|k}^{S_{1}}\right)}^{-1}{\hat{x}}_{i,k|k}^{S_{1}}+{\left(P_{j,k|k}^{S_{2}}\right)}^{-1}{\hat{x}}_{j,k|k}^{S_{2}}-{\left(P_{k|k}^{c}\right)}^{-1}{\hat{x}}_{k|k}^{c} \end{array}$$

and the scaling factor is:

(C9) $$c_{ij,k|k}=\exp\left(\zeta_{i,k|k}^{S_{1}}+\zeta_{j,k|k}^{S_{2}}-\zeta_{k|k}^{c}-\zeta_{ij,k|k}\right)$$

with:

(C10) $$\begin{array}{l} \zeta_{i,k|k}^{S_{1}}=-\frac{1}{2}\left(d\log2\pi -\log\left|{\left(P_{i,k|k}^{S_{1}}\right)}^{-1}\right|+{\left({\hat{x}}_{i,k|k}^{S_{1}}\right)}^{T}{\left(P_{i,k|k}^{S_{1}}\right)}^{-T}{\hat{x}}_{i,k|k}^{S_{1}}\right)\\ \zeta_{j,k|k}^{S_{2}}=-\frac{1}{2}\left(d\log2\pi -\log\left|{\left(P_{j,k|k}^{S_{2}}\right)}^{-1}\right|+{\left({\hat{x}}_{j,k|k}^{S_{2}}\right)}^{T}{\left(P_{j,k|k}^{S_{2}}\right)}^{-T}{\hat{x}}_{j,k|k}^{S_{2}}\right)\\ \zeta_{k|k}^{c}=-\frac{1}{2}\left(d\log2\pi -\log\left|{\left(P_{k|k}^{c}\right)}^{-1}\right|+{\left({\hat{x}}_{k|k}^{c}\right)}^{T}{\left(P_{k|k}^{c}\right)}^{-T}{\hat{x}}_{k|k}^{c}\right)\\ \zeta_{ij,k|k}=-\frac{1}{2}\left(d\log2\pi -\log\left|P_{ij,k|k}^{-1}\right|+{\hat{x}}_{ij,k|k}^{T}P_{ij,k|k}^{-T}{\hat{x}}_{ij,k|k}\right) \end{array}$$

## References

[1] Reali F., Palmerini G.B., Farina A., Graziano A., Giompapa S. (2013). Parametric analysis of ballistic target-tracking problem by multiple model approach. IET Radar Sonar Navig..

[2] Benavoli A., Chisci L., Farina A. (2007). Tracking of a Ballistic Missile with A-Priori Information. IEEE Trans. Aerosp. Electron. Syst..

[3] Farina A., Ristic B., Benvenuti D. (2002). Tracking a ballistic target: comparison of several nonlinear filters. IEEE Trans. Aerosp. Electron. Syst..

[4] Saulson B., Chang K.C. (2004). Nonlinear Estimation Comparison for Ballistic Missile Tracking. Opt. Eng..

[5] Dodin P., Minvielle P., Cadre J.P.L. (2007). Estimating the Ballistic Coefficient of a Re-Entry Vehicle. IET Radar Sonar Navig..

[6] Zhao Z., Chen H., Chen G., Kwan C., Li X.R. Comparison of Several Ballistic Target Tracking Filters. Proceedings of the American Control Conference.

[7] Farina A., Benvenuti D., Ristic B. Estimation Accuracy of a Landing Point of a Ballistic Target. Proceedings of the Fifth International Conference on Information Fusion.

[8] Ristic B., Farina A., Benvenuti D., Arulampalam M.S. (2003). Performance Bounds and Comparison of Nonlinear Filters for Tracking a Ballistic Object on Re-Enty. IEE Proc. Radar Sonar Navig..

[9] Julier S.J., Uhlmann J.K. (2004). Unscented Filtering and Nonlinear Estimation. Proc. IEEE.

[10] Singh N.K., Bhaumik S., Bhattacharya S. Tracking of Ballistic Target on Re-Entry Using Ensemble Kalman Filter. Proceedings of the Annual IEEE India Conference.

[11] Tseng C.H., Lin S.F., Jwo D.J. (2016). Fuzzy Adaptive Cubature Kalman Filter for Integrated Navigation Systems. Sensors.

[12] Zhu W., Wang W., Yuan G. (2016). An Improved Interacting Multiple Model Filtering Algorithm Based on the Cubature Kalman Filter for Maneuvering Target Tracking. Sensors.

[13] Barrios C., Motai Y., Huston D. (2016). Intelligent Forecasting Using Dead Reckoning with Dynamic Errors. IEEE Trans. Ind. Inf..

[14] Lee S.J., Motai Y., Choi H. (2013). Tracking Human Motion with Multichannel Interacting Multiple Model. IEEE Trans. Ind. Inf..

[15] Himberg H., Motai Y., Bradley A. (2013). A Multiple Model Approach to Track Head Orientation with Delta Quaternions. IEEE Trans. Cybern..

[16] Chang K., Chong C.Y., Mori S. (2010). Analytical and Computational Evaluation of Scalable Distributed Fusion Algorithms. IEEE Trans. Aerosp. Electron. Syst..

[17] Grime S., Durrant-Whyte H. (1994). Communication in Decentralized Systems. IFAC Control Eng. Pract..

[18] Hurley M.B. An Information Theoretic Justification for Covariance Intersection and Its Generalization. Proceedings of the Fifth International Conference on Information Fusion.

[19] Liggins M., Hall D., Llinas J. (2008). Handbook of Multisensor Data Fusion: Theory and Practice.

[20] Martin T.W., Chang K.C. A Distributed Data Fusion Approach for Mobile Ad Hoc Networks. Proceedings of the 8th International Conference on Information Fusion.

[21] Weng Y., Xiao W., Xie L. (2011). Diffusion-Based Em Algorithm for Distributed Estimation of Gaussian Mixtures in Wireless Sensor Networks. Sensors.

[22] Julier S.J. An Empirical Study into the Use of Chernoff Information for Robust, Distributed Fusion of Gaussian Mixture Models. Proceedings of the 9th International Conference on Information Fusion.

[23] Gunay M., Orguner U., Demirekler M. Approximate Chernoff Fusion of Gaussian Mixtures Using Sigma-Points. Proceedings of the 17th International Conference on Information Fusion (FUSION).

[24] Üney M., Clark D.E., Julier S.J. (2013). Distributed Fusion of PHD Filters via Exponential Mixture Densities. IEEE J. Sel. Top. Sign. Proces..

[25] Shin S.J. (2015). Re-Entry Vehicle Tracking with a New Multiple Model Estimation Applicable to Highly Non-Linear Dynamics. IET Radar Sonar Navig..

[26] Farrell W. (2008). Interacting Multiple Model Filter for Tactical Ballistic Missile Tracking. IEEE Trans. Aerosp. Electron. Syst..

[27] Bar-Shalom Y., Li X.R. (2001). Estimation with Applications to Tracking and Navigation.

[28] Li W., Jia Y. (2015). An Information Theoretic Approach to Interacting Multiple Model Estimation. IEEE Trans. Aerosp. Electron. Syst..

[29] Tian X., Bar-Shalom Y. On Algorithms for Asynchronous Track-to-Track Fusion. Proceedings of the 13th Conference on Information Fusion (FUSION).

[30] Hall D., Chong C.Y., Llinas J., Liggins M. (2012). Distributed Data Fusion for Network-Centric Operations.

[31] Chang K., Chong C.Y., Mori S. On Scalable Distributed Sensor Fusion. Proceedings of the 11th International Conference on Information Fusion.

[32] Runnalls A.R. (2007). Kullback-Leibler Approach to Gaussian Mixture Reduction. IEEE Trans. Aerosp. Electron. Syst..

[33] Bromiley P. Products and Convolutions of Gaussian Probability Density Functions. http://www.tina-vision.net/docs/memos/2003-003.pdf.

